# AHR and GPER mediate the stimulatory effects induced by 3-methylcholanthrene in breast cancer cells and cancer-associated fibroblasts (CAFs)

**DOI:** 10.1186/s13046-019-1337-2

**Published:** 2019-08-01

**Authors:** Francesca Cirillo, Rosamaria Lappano, Leonardo Bruno, Bruno Rizzuti, Fedora Grande, Rita Guzzi, Sara Briguori, Anna Maria Miglietta, Miki Nakajima, Maria Teresa Di Martino, Marcello Maggiolini

**Affiliations:** 1 Department of Pharmacy, Health and Nutritional Sciences, University of Calabria, 87036 Cosenza, Rende Italy; 2 Department of Biology, Ecology and Earth Sciences, University of Calabria, 87036 Rende, Italy; 3 CNR-NANOTEC, Licryl-UOS Cosenza and CEMIF. Cal and Department of Physics, University of Calabria, 87036 Rende, Italy; 4 Molecular Biophysics Laboratory, Department of Physics, University of Calabria, 87036 Rende, Italy; 5 Regional Hospital Cosenza, 87100 Cosenza, Italy; 6 Drug Metabolism and Toxicology, WPI Nano Life Science Institute, Kanazawa University, Kakuma-machi, Kanazawa, 920-1192 Japan; 7 Department of Experimental and Clinical Medicine, Magna Graecia University, 88100 Catanzaro, Italy

**Keywords:** 3-methylcholanthrene, GPER, AHR, CYP1B1, Breast Cancer, Cancer-associated fibroblasts

## Abstract

**Background:**

The chemical carcinogen 3-methylcholanthrene (3MC) binds to the aryl hydrocarbon receptor (AHR) that regulates the expression of cytochrome P450 (CYP) enzymes as CYP1B1, which is involved in the oncogenic activation of environmental pollutants as well as in the estrogen biosynthesis and metabolism. 3MC was shown to induce estrogenic responses binding to the estrogen receptor (ER) α and stimulating a functional interaction between AHR and ERα. Recently, the G protein estrogen receptor (GPER) has been reported to mediate certain biological responses induced by endogenous estrogens and environmental compounds eliciting an estrogen-like activity.

**Methods:**

Molecular dynamics and docking simulations were performed to evaluate the potential of 3MC to interact with GPER. SkBr3 breast cancer cells and cancer-associated fibroblasts (CAFs) derived from breast tumor patients were used as model system. Real-time PCR and western blotting analysis were performed in order to evaluate the activation of transduction mediators as well as the mRNA and protein levels of CYP1B1 and cyclin D1. Co-immunoprecipitation studies were performed in order to explore the potential of 3MC to trigger the association of GPER with AHR and EGFR. Luciferase assays were carried out to determine the activity of CYP1B1 promoter deletion constructs upon 3MC exposure, while the nuclear shuttle of AHR induced by 3MC was assessed through confocal microscopy. Cell proliferation stimulated by 3MC was determined as biological counterpart of the aforementioned experimental assays. The statistical analysis was performed by ANOVA.

**Results:**

We first ascertained by docking simulations the ability of 3MC to interact with GPER. Thereafter, we established that 3MC activates the EGFR/ERK/c-Fos transduction signaling through both AHR and GPER in SkBr3 cells and CAFs. Then, we found that these receptors are involved in the up-regulation of CYP1B1 and cyclin D1 as well as in the stimulation of growth responses induced by 3MC.

**Conclusions:**

In the present study we have provided novel insights regarding the molecular mechanisms by which 3MC may trigger a physical and functional interaction between AHR and GPER, leading to the stimulation of both SkBr3 breast cancer cells and CAFs. Altogether, our results indicate that 3MC may engage both GPER and AHR transduction pathways toward breast cancer progression.

**Electronic supplementary material:**

The online version of this article (10.1186/s13046-019-1337-2) contains supplementary material, which is available to authorized users.

## Background

Polycyclic aromatic hydrocarbons (PAHs) are a large group of organic pollutants widely distributed within the environment [1]. Numerous studies have associated the exposure to PAHs with an increased risk to develop various types of tumor as skin, lung, liver and breast cancers [2–7]. On the basis of these observations corroborated by the mutagenic and carcinogenic effects elicited by PAHs in mouse models, the International Agency for Research on Cancer has indicated that several PAHs are potential human carcinogens [8–10]. As the oncogenic activation of PAHs is concerned, it involves the cytochrome P450 (CYP)-dependent biotransformation of these agents in reactive diol-epoxides that bind to and induce the DNA damage [11–15]. Among the CYP family members, especially CYP1A1, CYP1A2 and CYP1B1 are responsible for the carcinogenic activation of many PAHs like 3-methylcholanthrene (3MC) [11–15]. In addition, CYP1B1 has been involved in both estrogen biosynthesis and metabolism through which DNA damaging chemicals (for instance 4-hydroxyestradiol) can be generated [11, 12, 16–18]. Considering that CYP1B1 contributes to both the carcinogenic activation of environmental chemicals and the bio-transformation of endogenous estrogens, its role in the initiation and progression of hormone-dependent malignancies, including breast cancer, has been suggested [12, 19]. CYP1B1 and other CYP enzymes are regulated by the aryl hydrocarbon receptor (AHR) [20], which is a ligand-activated transcription factor involved in the tumor-promoting properties of different environmental contaminants like PAHs [21–23]. Worthy, it has been also demonstrated that upon activation by ligands, including 3MC, AHR triggers stimulatory effects in cancer cells through a functional cooperation with the estrogen receptor α (ERα) and other transduction pathways as growth factor receptors [24–30]. Surprisingly, other studies provided evidence regarding the ability of 3MC to induce the expression of estrogen target genes via a direct binding to ERα in various tumor cell contexts [31–34].

The recent identification of a seven-transmembrane receptor namely GPER (G protein estrogen receptor, formerly GPR30), which is able to mediate the estrogen action, has suggested a further mechanism through which estrogenic compounds may induce relevant biological responses in both tumor cells and important components of the surrounding microenvironment like cancer associated fibroblasts (CAFs) [35–44]. GPER activation has been shown to trigger diverse transduction pathways as epidermal growth factor receptor (EGFR), mitogen-activated protein kinase (MAPK), cyclic AMP (cAMP) and calcium mobilization [28, 45, 46]. These effects mediated by GPER may lead to a peculiar gene signature that facilitates cancer cell growth and migration [35]. In the framework of the aforementioned findings, we have also assessed that estrogenic GPER signaling triggers CYP1B1 expression toward breast cancer progression [47].

Considering the involvement of GPER in the multifaceted actions exerted by estrogens and environmental contaminants [39, 40, 48–50], in the present study we aimed to provide novel insights on the ability of 3MC to elicit stimulatory effects through both AHR and GPER in breast cancer cells and CAFs.

## Methods

### Molecular docking

The structure of GPER was built by using GPCR-I-TASSER, which is an algorithm specifically designed to model G protein-coupled receptors [51]. The resulting conformation was a seven-helix structure, in agreement with previous predictions [52, 53], with the exception of the first 50 amino acid residues in the N-terminal regions that did not make part of the helix bundle core and therefore are not included in the transmembrane region. The protein conformation was refined through molecular dynamics (MD) simulations performed with the GROMACS package [54]. 3MC is a benz [*a*] antracene derivative characterized by a rigid polycondensed cyclic structure, which lacks any flexibility due to the absence of rotatable bonds involving non-hydrogen atoms. Classical molecular docking subsumes apolar hydrogens into the carbon atoms they are attached to, and adapts the ligand to the receptor through a search consisting in rotations around chemical bonds. Thus, this technique may only provide a poor prediction of the binding to GPER, and it was only applied to obtain starting models of the ligand/protein complex subsequently refined by further MD simulations. In particular, the same protocol was applied to test the binding of 3MC to GPER along with three known ligands: the agonists 17β-estradiol (E2) and G-1, and the antagonist G15. AutoDock Vina [55] was used to predict the initial position of 3MC bound to GPER. Both the ligand and the protein were prepared through AutoDock Tools [56] in order to convert the structures and merge apolar hydrogens. A volume of 32 Å × 44 Å × 36 Å was identified within GPER, including any potential cavity for the ligand binding, and very high exhaustiveness was employed in the roto-translation of 3MC. The best ten docking poses were clustered to reduce the number of similar binding modes (within a cut-off distance < 3 Å), resulting in four 3MC distinct initial locations. MD simulations of the molecular complexes were carried out for each starting pose by using the AMBER ff99SB-ILDN force field [57] for the protein and GAFF [58] for the ligand. Each run was carried out for 10 ns in the isobaric-isothermal ensemble in explicit water, with Cl-counterions added to obtain an overall neutral system. Other simulation conditions were as previously described for similar protein-ligand complexes [59, 60]. The system was first equilibrated for 2.5 ns and structures were afterwards sampled every 0.5 ns to evaluate the binding energy and the ligand location. The affinity was assessed by using the AutoDock Vina scoring function [55] without any further search (i.e. using a score-only evaluation).

### Reagents and drugs

3-Methylcholanthrene (3MC) and CH223191 (1-Methyl-*N*-[2-methyl-4-[2-(2-methylphenyl)diazenyl]phenyl-1*H*-pyrazole-5-carboxamide) were purchased from Sigma-Aldrich (Milan, Italy). (3aS,4R,9bR)-4-(6-bromo-1,3-benzodioxol-5-yl)-3a,4,5,9b-3*H*-cyclopenta [*c*] quinolone (G15) and 1-[2,(3,5-dimethoxyphenyl) ethenyl]-2,4-dimethoxybenzene (TMS) were obtained from Tocris Bioscience (Space, Milan, Italy). Mithramycin A (MTM A) was purchased from Abcam (Euroclone, Milan, Italy). CH223191 and G15 were dissolved in dimethyl sulfoxide (DMSO), 3MC in toluene and MTM A in ethanol. All reagents were used at concentrations previously reported [61–63].

### Cell cultures

SkBr3 breast cancer cells were provided by ATCC (Manassas, VA, USA), used less than 6 months after resuscitation, routinely tested and authenticated according to the ATCC suggestions. SkBr3 cells were maintained in RPMI-1640 (Life Technologies, Milan, Italy) without phenol red, supplemented with 10% fetal bovine serum (FBS) and 100 μg/ml penicillin/streptomycin (Life Technologies, Milan, Italy). CAFs were obtained as previously described [47] from 5 invasive ductal breast carcinomas and pooled for the subsequent studies. Briefly, specimens were cut into 1–2 mm diameter pieces, placed in a digestion solution consisting of 400 IU collagenase, 100 IU hyaluronidase, 10% serum, antibiotics and antimycotics, and incubated overnight at 37 °C. After centrifugation at 90×g for 2 min, supernatant containing fibroblasts was centrifuged at 485×g for 8 min; the pellet obtained was suspended in Medium 199 and Ham’s F12 mixed 1:1 (supplemented with 10% FBS and 100 μg/ml penicillin/streptomycin). CAFs were then expanded into 10-cm Petri dishes and stored as cells passaged for three population doublings within total 7 to 10 days after tissue dissociation. Primary cell cultures of fibroblasts were characterized by immunofluorescence with human anti-vimentin (V9) and human anti-cytokeratin 14 (LL001) (Santa Cruz Biotechnology, DBA, Milan, Italy). FAPα antibody (H-56; Santa Cruz Biotechnology, DBA, Milan, Italy) was used to characterize activated fibroblasts (Additional file 1). We used CAFs passaged for up to ten population doublings for the experiments, to minimize clonal selection and culture stress, which could occur during extended tissue culture. All cell lines were grown in a 37 °C incubator with 5% CO_2_ and switched to medium without serum and phenol red the day before treatments to be processed for immunoblot and RT-PCR assays.

### Gene expression studies

Total RNA was extracted and cDNA was synthesized by reverse transcription as previously described [64]. The expression of selected genes was quantified by real-time PCR using platform Quant Studio7 Flex Real-Time PCR System (Life Technologies). Gene-specific primers were designed using Primer Express version 2.0 software (Applied Biosystems). For CYP1B1, c-Fos, cyclin D1 and the ribosomal protein 18S, which was used as a control gene to obtain normalized values, the primers were: 5′-TGTGCCTGTCACTATTCCTCATG-3′ (CYP1B1 forward) and 5′-GGGAATGTGGTAGCCCAAGA-3′ (CYP1B1 reverse); 5′-CGAGCCCTTTGATGACTTCCT-3′ (c-Fos forward) and 5′-GGAGCGGGCTGTCTCAGA-3′ (c-Fos reverse); 5′-GTCTGTGCATTTCTGGTTGCA-3′ (cyclin D1 forward) and 5′-GCTGGAAACATGCCGGTTA-3′ (cyclin D1 reverse); 5′-TGGTCAGTGCCTTGTTGGATG-3′ (ERα forward) and 5′-TGTCTTGCCAGGTTGGTCAGTAAG-3′ (ERα reverse); 5′- ACACACCTGGGTGGACACAA-3′ (GPER forward) and 5′-GGAGCCAGAAGCCACATCTG-3′ (GPER reverse); 5′-GGCGTCCCCCAACTTCTTA-3′ (18S forward) and 5′-GGGCATCACAGACCTGTTATT-3′ (18S reverse). Assays were performed in triplicate and the results were normalized for 18S expression and then calculated as fold induction of RNA expression.

### Western blotting analysis

Cells were grown in 10-cm dishes, exposed to treatments and then lysed in 500 μL of 50 mmol/L NaCl, 1.5 mmol/L MgCl_2_, 1 mmol/L EGTA, 10% glycerol, 1% Triton X-100, 1% sodium dodecyl sulfate (SDS), and a mixture of protease inhibitors containing 1 mmol/L aprotinin, 20 mmol/L phenylmethylsulfonyl fluoride and 200 mmol/L sodium orthovanadate. Protein concentration was determined using Coomassie (Bradford) protein reagent according to the manufacturer’s recommendations (Life Technologies, Milan, Italy). Equal amounts of whole protein extract were resolved on a 10% SDS-polyacrylamide gel, transferred to a nitrocellulose membrane (Amersham Biosciences, Sigma-Aldrich, Milan, Italy), probed overnight at 4 °C with antibodies against CYP1B1 (TA339934) and cyclin D1 (TA801655) (purchased from OriGene Technologies, DBA, Milan, Italy), GPER (AB137479) (Abcam, Euroclone, Milan, Italy), AHR (D5S6H) (Cell Signalling technology, Euroclone, Milan, Italy), c-Fos (E8), pEGFR Tyr 1173 (sc-12,351), EGFR (1005), phosphorylated ERK1/2 (E-4), ERK2 (C-14) and β-actin (C-2) (purchased from Santa Cruz Biotechnology, DBA, Milan, Italy). Proteins were detected by horseradish peroxidase-linked secondary antibodies (Bio-Rad, Milan, Italy) and then revealed using the chemiluminescent substrate for western blotting Westar Nova 2.0 (Cyanagen, Biogenerica, Catania, Italy).

### Immunoprecipitation assay

After exposure to treatments, cells were washed and lysed using 500 μl RIPA buffer with protease inhibitors (1.7 mg/ml aprotinin, 1 mg/ml leupeptin, 200 mmol/liter phenylmethylsulfonyl fluoride, 200 mmol/liter sodium orthovanadate and 100 mmol/liter sodium fluoride). Samples were then centrifuged at 13,000 rpm for 10 min and protein concentrations were determined using Coomassie (Bradford) protein assay. Proteins (250 μg) were then incubated for 2 h with 900 μl of immunoprecipitation buffer with inhibitors, 2 μg of anti-GPER, anti-AHR or anti-EGFR antibodies and 20 μl of Protein A/G agarose immunoprecipitation reagent (Santa Cruz Biotechnology, DBA, Milan, Italy). Samples were then centrifuged at 13,000 rpm for 5 min at 4 °C to pellet beads. Pellets were washed four times with 500 μl of PBS and centrifuged at 13,000 rpm for 5 min at 4 °C. Supernatants were collected, resuspended in 20 μl RIPA buffer with protease inhibitors, 2X SDS sample buffer and heated to 95 °C for 5 min. Samples were then run on 10% SDS-PAGE, transferred to nitrocellulose and probed with primary antibodies. Western blot analysis and ECL detection were performed as described above.

### Immunofluorescence assay

Cells were grown on a cover slip, serum deprived for 18 h and exposed to treatments for 4 h, when required. Cells were then fixed in ice-cold methanol at room temperature for 10 min, permeabilized with 0.2% Triton X-100, washed three times with PBS and incubated with 1% BSA in PBS at room temperature for 1 h. After washing with PBS, cells were incubated with primary antibodies against AHR (D5S6H) (Cell Signalling technology, Euroclone, Milan, Italy), vimentin (V9), cytokeratin 14 (LL001) and FAPα (H-56) (Santa Cruz Biotechnology, DBA, Milan, Italy) (diluted in 1% BSA/PBS) at 4 °C for 18 h. After incubation, cells were washed three times with PBS and incubated with Alexa fluor conjugated secondary antibodies (Thermofisher Scientific, Milan, Italy) for 1 h at room temperature. Finally, cells were washed three times with PBS, incubated with DAPI (4′,6-diamidino-2-phenylindole) (1:1000) for 3 min and, after washing, immunofluorescence images for the characterization of CAFs were obtained by Cytation 3 Cell Imaging Multimode reader (BioTek) and analyzed using the software Gen5. As it concerns the evaluation of AHR nuclear translocation, immunofluorescence images were obtained by Leica inverted TCS SP8 confocal scanning laser microscope, with a 63X oil immersion objective.

### Gene silencing experiments

Cells were transfected using X-treme GENE 9 DNA Transfection Reagent (Roche Diagnostics, Sigma-Aldrich, Milan, Italy) for 24 h, prior to adding treatments, with control shRNA, shRNA for GPER (shGPER) [65] or shRNA for CYP1B1 (shCYP1B1, Santa Cruz Biotechnology, DBA, Milan, Italy).

### Bioinformatic tools and plasmids

The putative promoter sequence of CYP1B1 was retrieved from the National Center for Biotechnology Information (NCBI) (http://www.ncbi.nlm.nih.gov). The plasmid DN/c-Fos, which encodes a c-Fos mutant that heterodimerizes with c-Fos dimerization partners but does not allow DNA binding, was a kind gift from Dr. C. Vinson (NIH, Bethesda, MD, USA). pGL3-promoter plasmid containing the 5′-flanking region from − 2299 to + 25 with respect to the transcription initiation site (TIS) [66] of the CYP1B1 gene and CYP1B1 promoter deleted constructs containing fragments − 1652 to + 25, − 1022 to + 25, − 910 to + 25 with respect to TIS were generated as previously described [67]. All these constructs contain the half-ERE binging motif, as described in our previous work [47].

### Transfections and luciferase assays

Cells (1 × 10^5^) were plated into 24-well dishes with 500 μl/well of regular growth medium the day before transfection. Growth medium was replaced with medium lacking serum on the day of transfection, which was performed using X-tremeGene9 reagent, as recommended by the manufacturer (Roche Diagnostics), with a mixture containing 0.5 μg of each reporter plasmid and 1 ng of pRL-TK. After 8 h, the medium was replaced with fresh medium lacking serum and the cells were incubated for 18 h with treatments. Luciferase activity was then measured with the Dual Luciferase Kit (Promega, Milan, Italy) according to the manufacturer’s recommendations. Firefly luciferase activity was normalized to the internal transfection control provided by the Renilla luciferase activity. The normalized relative light unit values obtained from cells treated with vehicle (−) were defined as one-fold induction, relative to which the activity induced by treatments was calculated.

### Cell proliferation assays

Cells (1 × 10^5^) were seeded in 24-well plates in regular growth medium, washed once they had attached and then incubated in medium containing 5% charcoal-stripped FBS, transfected for 24 h (where appropriate) and then exposed to treatments. Transfection were renewed every 2 days and treatments every day. Cells were counted on day 5 using the Countess Automated Cell Counter, as recommended by the manufacturer’s protocol (Life Technologies, Milan, Italy).

For spheroid generation, 100 μl/well of SkBr3 cell suspensions (1 × 10^4^) were dispensed into 2% agar-coated 96-well plates. Three days after seeding, tumor spheroids (a single spheroid per well) were exposed to treatments and a 50% medium and treatment replenishment was performed every 2 days. Images were obtained on day 20 using a conventional inverted microscope, thereafter cell number per spheroid was determined by trypsinizing three different spheroids, mixing the cell suspension with trypan blue and counting the number of viable cells. The total number of cells obtained was divided by the number of trypsinized spheroids.

### Statistical analysis

Statistical analysis was done using ANOVA followed by Newman–Keuls’ testing to determine differences in means. *P* < 0.05 was considered as statistically significant.

## Results

### Interaction between GPER and 3MC in molecular dynamics and docking simulation

**Fig. 1:**
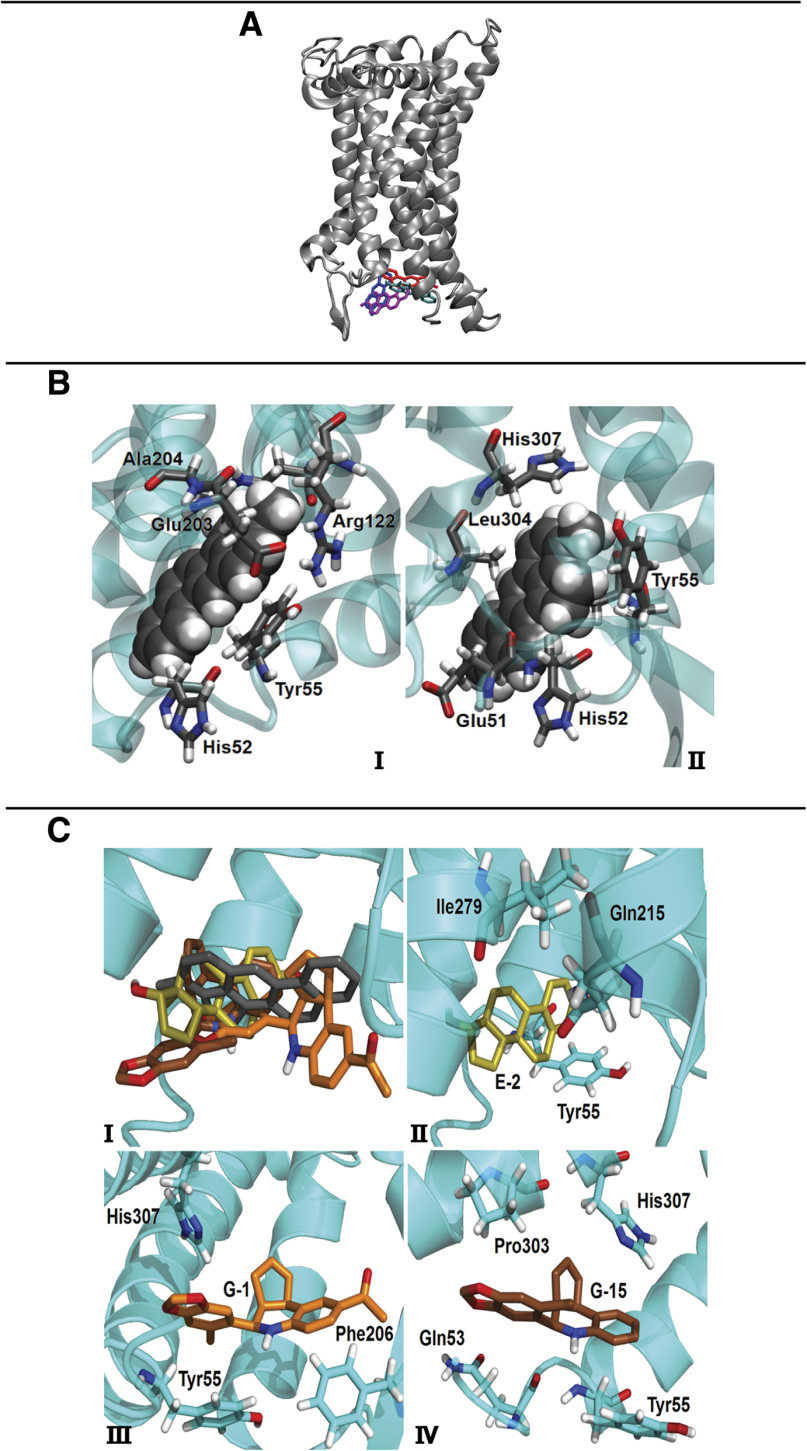
Docking poses of 3MC bound to GPER*.* **a** Four best poses selected by clustering the binding modes obtained in the docking simulations are shown. The binding affinity decreases in the following order: magenta → cyan → blue → red. The mobile N-terminal region of GPER (first 50 amino acids residues) is not presented. **b** Two representative binding modes of 3MC (in van der Waals representation) to GPER obtained in distinct MD runs are shown: **(I)** simulation S-4 and S-1, and **(II)** simulation S-2. Binding energies, as estimated with the scoring function of AutoDock Vina (see Materials and Methods), are similar in the two cases: − 8.3 ± 1.0 and − 8.2 ± 0.6 kcal/mol, respectively. Key protein residues in the binding pockets are also evidenced. **c** Binding modes of 3MC (gray), E2 (yellow), G-1 (orange) and G15 (brown) are indicated cumulatively in **(I).** Details of the protein residues anchoring the ligands to GPER are shown for E2 **(II)**, G-1 **(III)** and G15 **(IV)**

Given that several environmental compounds may exert pleiotropic effects through GPER [39, 40, 48, 50] and considering that 3MC is able to bind to ERα [31, 68], we aimed to provide new insights into the potential of 3MC to interact with GPER. Performing docking calculations in order to predict the 3MC-GPER complex, we evidenced binding modes with affinity scores ranging between − 7.8 and − 6.9 kcal/mol. In particular, we noticed 4 separate poses **(**Fig. 1**)** that were simulated in distinct molecular dynamics (MD) runs. The binding affinity was estimated on the equilibrated system to allow the molecule to adapt within the protein cavity emerging from the transmembrane region. A number of observations can be drawn from these data. First, there is no clear correlation between the binding scores obtained in the docking calculations and the ones estimated from the MD simulations. This indicates that the sole use of molecular docking to assess the binding energy of 3MC to GPER gives poor predictions of the affinity, as it could be expected for such a rigid ligand. Second, the most favorable binding modes during the MD simulations show in general a higher affinity toward the receptor compared to the corresponding docking poses (with the exception of simulation S-3; see also below). This observation further suggests to take into account the dynamics of the protein matrix to provide the ligand accommodation. The average value obtained in simulation gives an accurate prediction of the binding affinity of 3MC towards GPER. The docking scores calculated are consistent with the binding of 3MC in the pocket of GPER with good affinities (up to − 8.3 ± 1.0 kcal/mol), which would correspond to dissociation constants in the low micromolar range. The only exception was obtained in the simulation S-3, which reproduced at most a weak binding location of 3MC. Standard deviations from the average values of the binding affinities were in all cases ≤1 kcal/mol, consistent with the variations that could be expected due to thermal agitation of the ligand within the binding pocket. Visual inspections of the structures of complex sampled in the MD simulations gave further details on the binding locations of 3MC. As shown in Fig. 1b, the Tyr55 and His52 are key residues within the GPER site for the 3MC binding, allowing the accommodation of the ligand through interactions with their side chain ring and backbone group, respectively. In the binding position, 3MC may promote local deformations of the protein structure through two distinct mechanisms. One consists in bringing closer the β-hairpin between the helices H4 and H5, hence forming further interactions with it (simulations S-4 and S-1, Fig. 1b panel I). Alternatively, 3MC encourages distortion in the central region of the GPER N-terminal α-helix (i.e., H1) and inserts between it and the adjacent helix H2 (simulation S-2, Fig. 1b panel II). To further support the aforementioned results, docking simulations were also performed with three known ligands of GPER: the agonists E2 and G-1 and the antagonist G15. As shown in Fig. 1c panel I, all ligands (including 3MC) occupy the same binding pocket within GPER and differ only for details in their binding modes. The anchoring locations are in agreement with previous studies [52, 53, 69] that identified key GPER residues involved in the ligand association. For instance, Ile279 **(**Fig. 1c panel II) was already reported as a residue crucial for the binding of E2 [53, 69] and Phe206/His307 **(**Fig. 1c panel III) were found to facilitate the binding of G-1 [52, 53]. The binding energies of E2, G-1 and G15 (Fig. 1c panels II, III, IV) varied in the range between − 8.7 and − 7.8 kcal/mol, suggesting that 3MC may mimic these ligands to bind GPER, although with a slightly lower specificity and affinity. On the basis of our MD results, 3MC may act as a ligand of GPER occupying at least in two binding modes the same pocket identified in previous computational studies. The predicted affinity of 3MC (− 8.3 kcal/mol) is comparable to the binding energies obtained for other known ligands of GPER, although the variability in the anchoring location lead to a lower specificity.

### 3MC triggers the EGFR/ERK transduction pathway and c-fos expression through both AHR and GPER in breast cancer cells and CAFs

**Fig. 2:**
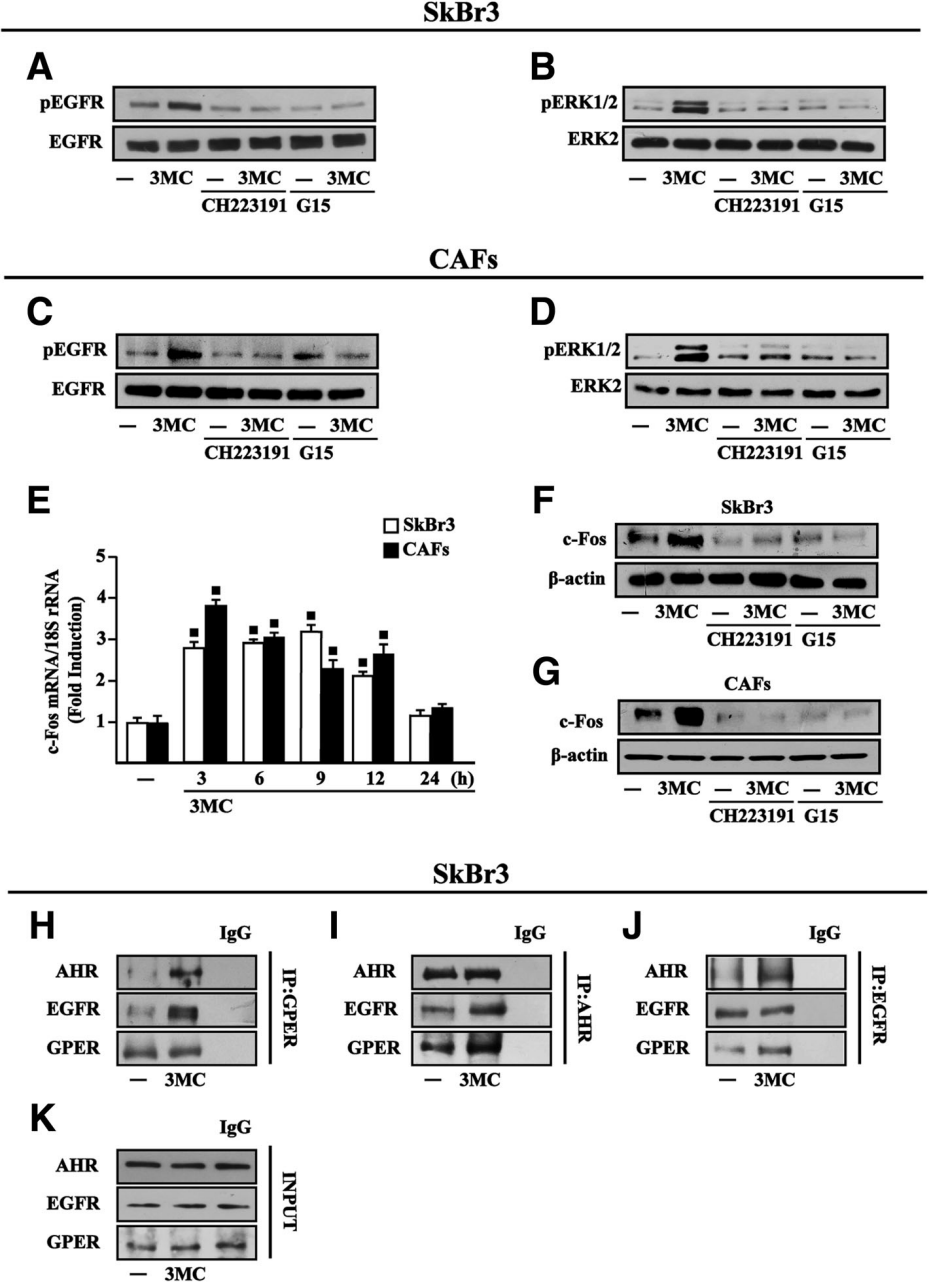
AHR and GPER are involved in the activation of the EGFR/ERK1/2/c-Fos transduction pathway by 3MC. Phosphorylation of EGFR (**a**, **c**) and ERK1/2 (**b**, **d**) in SkBr3 cells and CAFs treated for 15 min with vehicle (−) or 1 μM 3MC alone or in combination with either 1 μM AHR inhibitor CH223191 or 100 nM GPER antagonist G15, as indicated. EGFR and ERK2 serve as loading controls for pEGFR and pERK1/2, respectively. Results shown are representative of three independent experiments. **e** 1 μM 3MC induces the mRNA expression of c-Fos in SkBr3 cells and CAFs, as indicated. Data obtained by real-time PCR in three independent experiments performed each in triplicate were normalized to 18S expression and shown as fold changes of c-Fos expression upon treatment with 3MC respect to cells treated with vehicle (−). (■) indicates *P* < 0.05 for cells receiving treatments versus vehicle (−). Evaluation of c-Fos protein levels in SkBr3 cells (**f**) and CAFs (**g**) upon a 6 h treatment with vehicle (−) and 1 μM 3MC alone or in combination with either 1 μM AHR inhibitor CH223191 or 100 nM GPER antagonist G15. β-actin serves as a loading control. **h**-**j** Co-immunoprecipitation studies performed in SkBr3 cells treated with vehicle (−) or 1 μM 3MC for 1 h, as indicated. Cell lysates were immunoprecipitated with either anti-GPER (**h**), or anti-AHR (**i**) or anti-EGFR (**j**) antibodies. Immunocomplexes were analyzed by immunoblot with antibodies against the indicated proteins. In control samples, nonspecific IgG was used instead of the primary antibody. **k** Total lysates (input) were evaluated as control. Results shown are representative of at least two independent experiments

Previous studies have shown that AHR interacts with diverse growth factor transduction pathways in cancer cells [25, 70, 71]. In particular, it has been demonstrated that certain AHR ligands stimulate rapid kinase activation, gene expression changes and growth effects in cancer cells through a cross-talk between AHR and EGFR/ERK-mediated signaling [25, 72]. On the basis of these data and considering that also GPER activation triggers the EGFR/ERK transduction cascade in cancer cells [45], we first assessed that the rapid EGFR and ERK activation induced by 3MC is prevented either by the AHR inhibitor CH223191 or the GPER antagonist G15 in both ERα negative and GPER positive (data not shown) SkBr3 breast cancer cells and CAFs **(**Fig. 2a-d**)**. In accordance with our previous findings showing that the EGFR/ERK transduction pathway regulates several GPER target genes including c-Fos [35] and reminiscing previous data showing that 3MC stimulates the expression of c-Fos via AHR [24, 73, 74], we established that 3MC induces c-Fos mRNA (Fig. 2e) and protein levels (Fig. 2f-g) in SkBr3 cells and CAFs. Remarkably, the c-Fos protein expression upon 3MC-stimulation was no longer observed either using the AHR inhibitor CH223191 or the GPER antagonist G15 in both cell contexts (Fig. 2f-g). Previous studies have demonstrated that a physical and functional interaction of GPER with steroid (for instance, ERα and MR) and growth factor receptors (for instance, EGFR and IGF-IR) may be involved in cell cycle progression and tumor growth [75–77]. On the basis of these findings and previous observations showing that AHR plays a role in cancer cell proliferation by interacting with growth factor receptors including EGFR (70), we explored whether a physical association of GPER with AHR and EGFR may occur in SkBr3 cells upon exposure to 3MC. Therefore, cell lysates were immunoprecipitated with either anti-GPER (Fig. 2h) or anti-AHR (Fig. 2i) or anti-EGFR (Fig. 2j) antibodies. Each immunoprecipitate was analysed by immunoblot with either anti-GPER, anti-AHR or anti-EGFR antibodies. As shown in Fig. 2 (panels 2 h-k), 3MC was able to trigger the co-immunoprecipitation of GPER, AHR and EGFR, thus generating a ternary complex assembly of these receptors in SkBr3 breast cancer cells toward their functional cooperation.

### 3MC induces AHR nuclear translocation and CYP1B1 e*xpression through AHR and GPER*

**Fig. 3:**
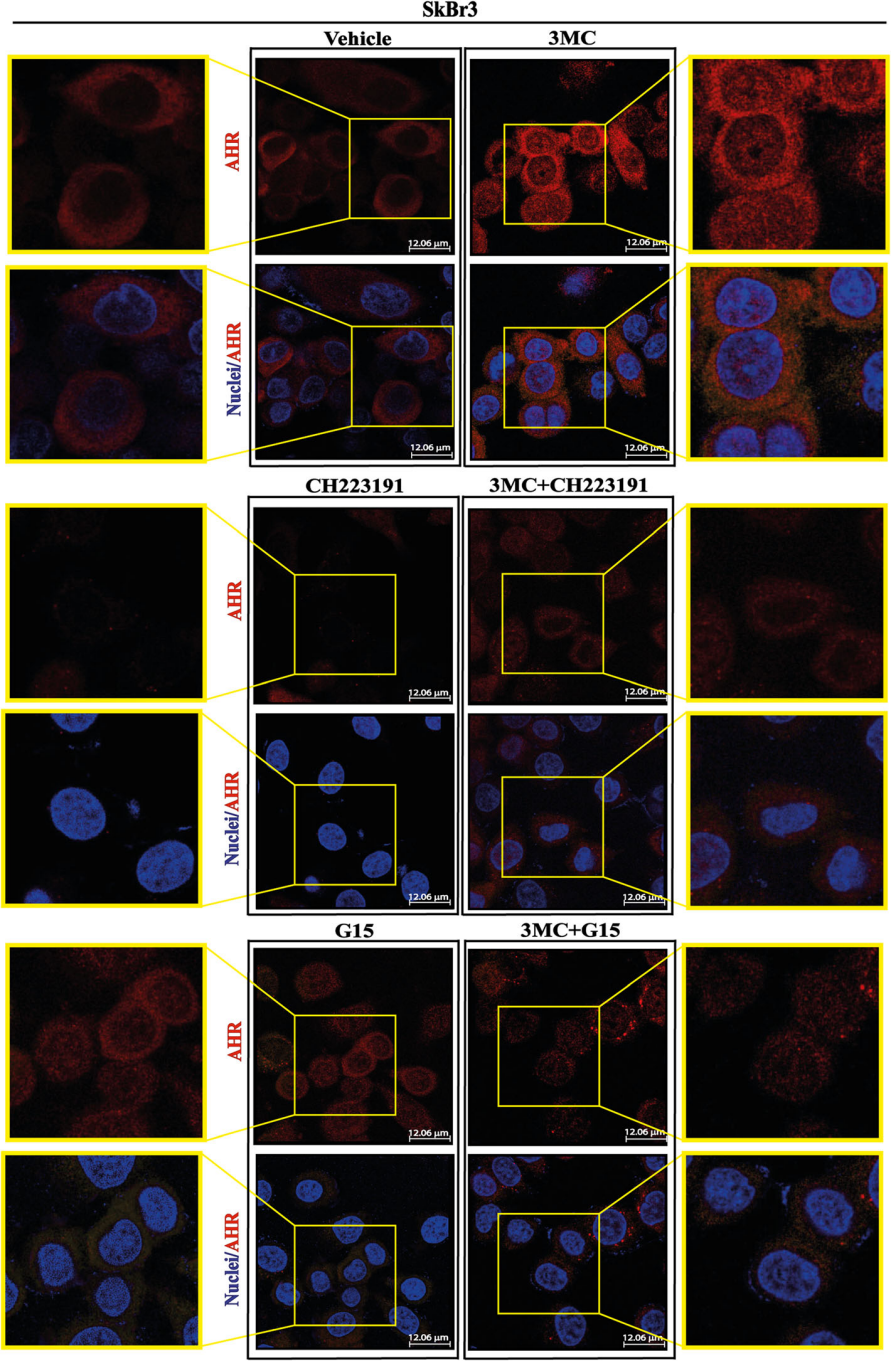
AHR nuclear translocation induced by 3MC is prevented by the AHR inhibitor CH223191 and the GPER antagonist G15. The AHR nuclear translocation in SkBr3 cells exposed for 1 h to 1 μM 3MC is prevented by 1 μM AHR inhibitor CH223191 and 100 nM GPER antagonist G15. Merge panels depict the overlap of the two fluorophores used to assess the nuclear translocation of AHR. Red signal: AHR. Blue signal: Nuclei. Images shown are representative of ten random fields from three independent experiments. Scale bar 12.06 μm

**Fig. 4:**
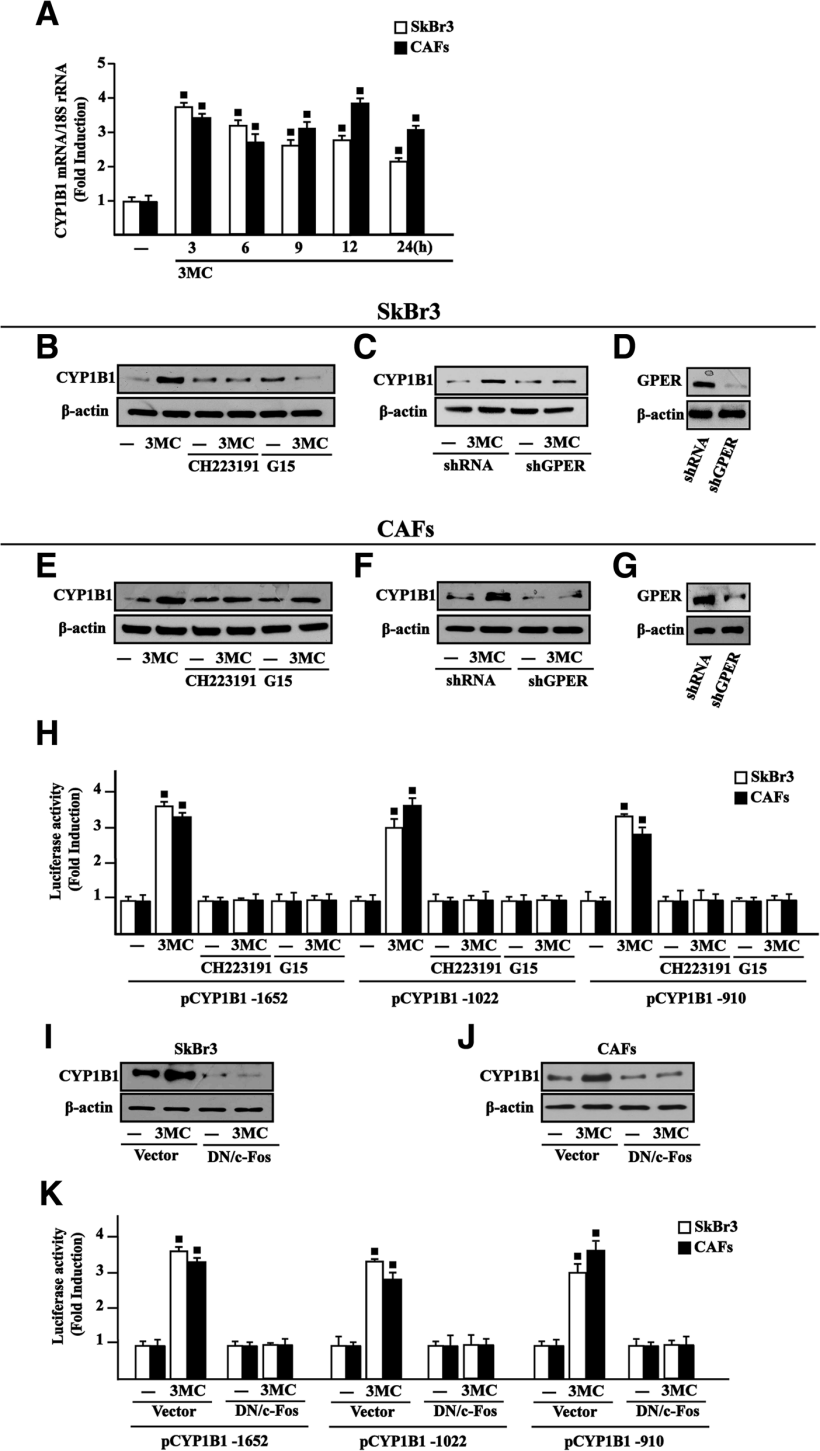
AHR and GPER are involved in CYP1B1 induction by 3MC. **a** 1 μM 3MC induces the mRNA expression of CYP1B1 in SkBr3 breast cancer cells and CAFs, as indicated. Data obtained by real-time PCR in three independent experiments performed in triplicate were normalized to the expression of 18S and shown as fold changes of CYP1B1 expression upon treatment with 3MC with respect to cells treated with vehicle (−). Evaluation of CYP1B1 protein levels in SkBr3 cells (**b**) and CAFs (**e**) upon treatment for 6 h with vehicle (−), 1 μM 3MC alone and in combination with either 1 μM AHR inhibitor CH223191 or 100 nM GPER antagonist G15. The up-regulation of CYP1B1 protein levels induced by a 6 h treatment with 1 μM 3MC is abrogated in SkBr3 cells (**c**) and CAFs (**f**) transfected for 24 h with shGPER. **d**, **g** Efficacy of GPER silencing. β-actin serves as a loading control. Results shown are representative of three independent experiments. **h** Luciferase activities of CYP1B1 promoter constructs in SkBr3 cells and CAFs treated for 18 h with vehicle (−) and 1 μM 3MC alone and in combination with either 1 μM AHR inhibitor CH223191 or 100 nM GPER antagonist G15, as indicated. CYP1B1 protein levels in SkBr3 cells (**i**) and CAFs (**j**) transfected for 18 h with a vector or DN/c-Fos construct and then treated for 6 h with vehicle (−) and 1 μM 3MC, as indicated. β-actin serves as a loading control. Results shown are representative of three independent experiments. **k** Luciferase activities of CYP1B1 promoter plasmids in SkBr3 cells and CAFs transfected for 8 h with CYP1B1 constructs, a vector or DN/c-Fos construct and then treated for 18 h with vehicle (−) and 1 μM 3MC. The luciferase activities were normalized to the internal transfection control and values of cells receiving vehicle (−) were set as 1-fold induction upon which the activities induced by treatments were calculated. Each column represents the mean ± SD of three independent experiments, each performed in triplicate. (■) indicates *P* < 0.05 for cells receiving treatments versus vehicle (−)

AHR mainly localizes within cell nuclei upon the binding to 3MC [20, 78]. In this regard, we found that the nuclear shuttle of AHR induced by 3MC in SkBr3 breast cancer cells is prevented either in the presence of the AHR inhibitor CH223191 or using the GPER antagonist G15 (Fig. 3). On the basis of previous data showing that 3MC induces CYP1B1 expression in tumor cells through AHR [14, 26, 79], we aimed to evaluate whether GPER may be involved in the expression of CYP1B1 by 3MC. By real-time PCR we first ascertained that 3MC up-regulates CYP1B1 mRNA levels in both SkBr3 cells and CAFs (Fig. 4a). Then, we determined that CYP1B1 protein expression upon treatment with 3MC is abolished using either the AHR inhibitor CH223191 or the GPER antagonist G15 (Fig. 4b, e) as well as silencing GPER expression (Fig. 4c-d, f-g). Similar results were obtained evaluating the transcriptional activation of the CYP1B1 promoter constructs transfected in SkBr3 cells and CAFs (Fig. 4h). Considering that in our previous study we established the involvement of c-Fos in CYP1B1 expression [47], we next found that the induction of CYP1B1 by 3MC is prevented transfecting SkBr3 cells and CAFs with the DN/c-Fos expression vector (Fig. 4i-j). Further supporting these findings, the transactivation of CYP1B1 promoter deletion constructs by 3MC was abolished in the presence of the DN/c-Fos construct (Fig. 4k). Overall, these data suggest that GPER contributes to the AHR-dependent up-regulation of CYP1B1 upon 3MC exposure in our model system.

### The growth responses triggered by 3MC occur through AHR and GPER

**Fig. 5:**
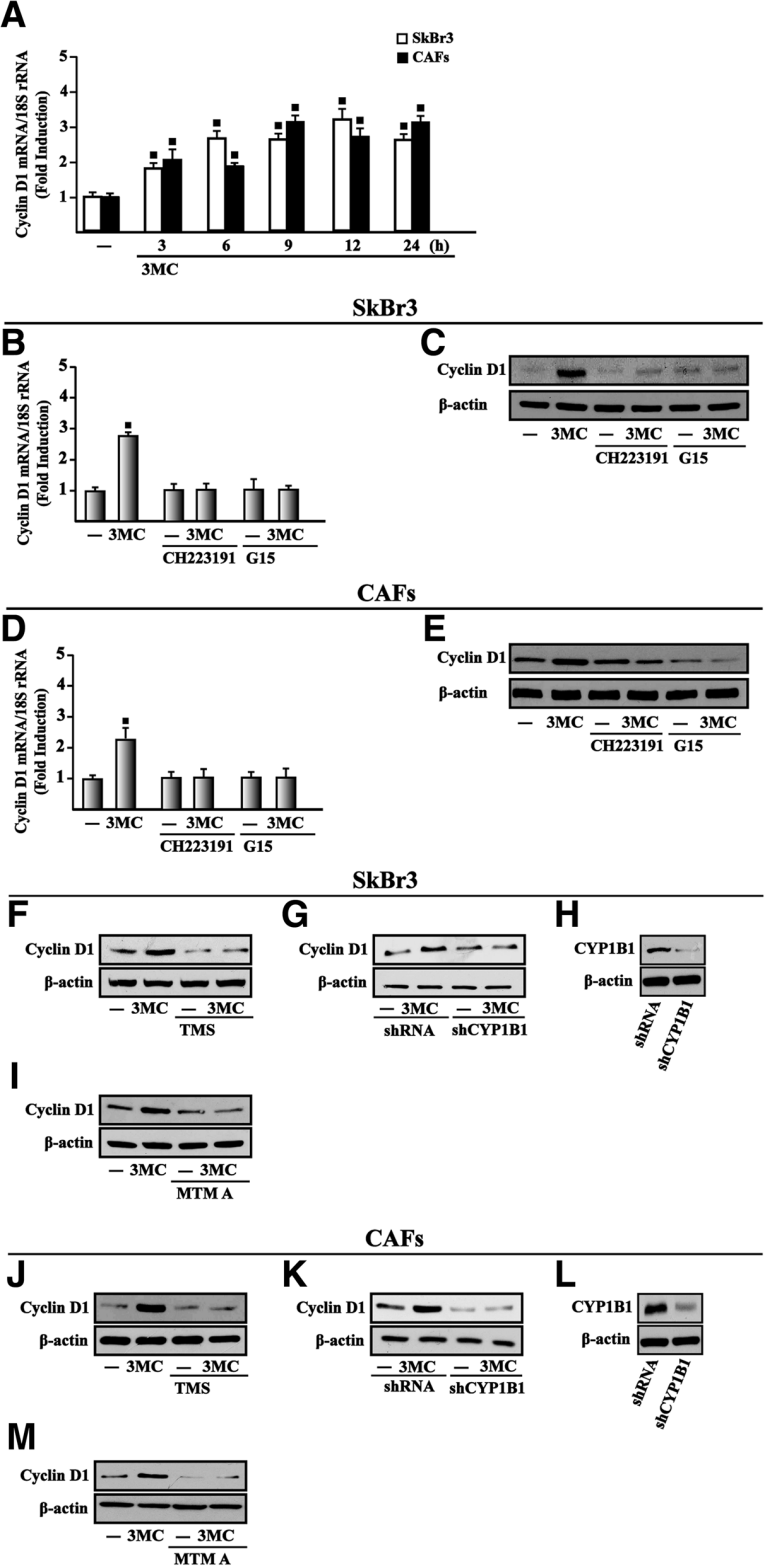
AHR and GPER are involved in the up-regulation of cyclin D1 by 3MC. **a** Cyclin D1 mRNA expression in SkBr3 cells and CAFs treated with vehicle (−) and 1 μM 3MC, as indicated. mRNA expression of cyclin D1 in SkBr3 cells (**b**) and CAFs (**d**) upon treatments for 18 h with vehicle (−) and 1 μM 3MC alone and in combination with either 1 μM AHR inhibitor CH223191 or 100 nM GPER antagonist G15, as indicated. Data obtained by real-time PCR in three independent experiments performed each in triplicate were normalized to 18S expression and shown as fold changes of Cyclin D1 expression induced by treatments with respect to cells treated with vehicle (−). Cyclin D1 protein levels in SkBr3 cells (**c**) and CAFs (**e**) upon treatments for 18 h with vehicle (−) and 1 μM 3MC alone and in combination with either 1 μM AHR inhibitor CH223191 or 100 nM GPER antagonist G15, as indicated. Cyclin D1 protein levels in SkBr3 cells (**f**) and CAFs (**j**) upon treatments for 18 h with vehicle (−) and 1 μM 3MC alone or in combination with 5 μM CYP1B1 activity inhibitor TMS. Cyclin D1 protein levels in SkBr3 cells (**g**) and CAFs (**k**) transiently transfected with shRNA or shCYP1B1 for 24 h and then treated for 18 h with vehicle (−) and 1 μM 3MC. **h, l** Efficacy of CYP1B1 silencing. Cyclin D1 protein levels in SkBr3 cells (**i**) and CAFs (**m**) treated for 18 h with vehicle (−) and 1 μM 3MC alone or in combination with 100 nM SP1 inhibitor Mithramycin A (MTM A). β-actin serves as a loading control. Results shown are representative of three independent experiments. (■) *P* < 0.05 for cells receiving treatments versus vehicle (−)

**Fig. 6:**
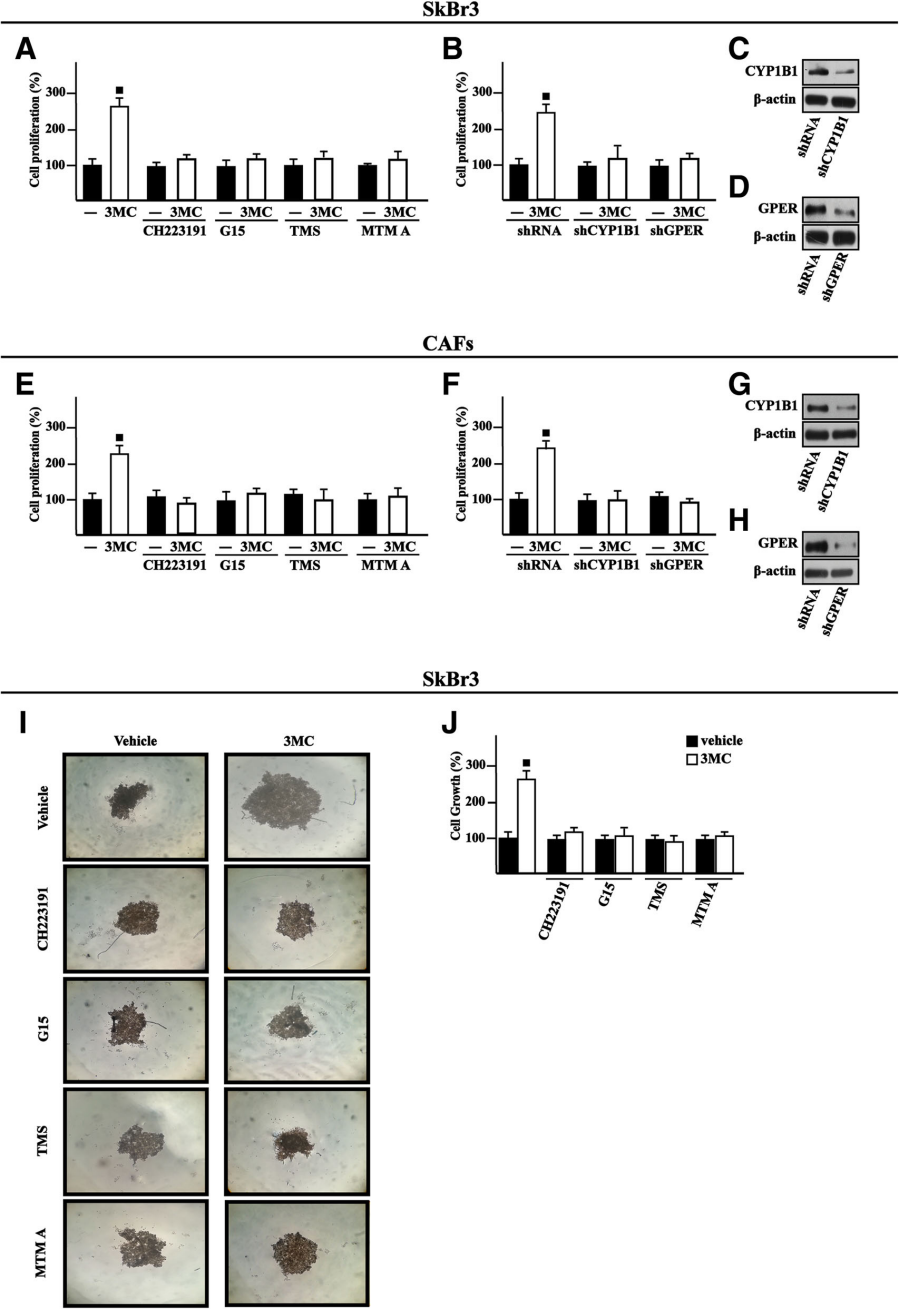
Transduction pathways involved in the proliferative effects triggered by 3MC. The proliferation of SkBr3 cells (**a**) and CAFs (**e**) induced by 1 μM 3MC is prevented by 1 μM AHR inhibitor CH223191, 100 nM GPER antagonist G15, 5 μM CYP1B1 inhibitor TMS and 100 nM SP1 antagonist mithramycin A (MTM A). The growth effects induced by 1 μM 3MC in SkBr3 cells (**b**) and CAFs (**f**) are prevented silencing either CYP1B1 or GPER expression. Cells were transfected every 2 days with shRNA, shCYP1B1 or shGPER, treated every day with ligands and then counted on day 5. Efficacy of the silencing of CYP1B1 (**c**, **g**) and GPER (**d**, **h**). β-actin serves as a loading control. Proliferation of cells treated with vehicle (−) was set as 100% upon which cell growth induced by treatments was calculated. Each data point is the mean ± SD of three independent experiments performed in triplicate. **i** Representative images of SkBr3 spheroids (a single spheroid per well) grown on agar-coated plates after 20 days treatment with vehicle (−) or 1 μM 3MC alone or in combination with 1 μM AHR inhibitor CH223191, 100 nM GPER antagonist G15, 5 μM CYP1B1 inhibitor TMS and 100 nM SP1 antagonist MTM A, as indicated. **j** Evaluation of SkBr3 cell growth upon treatments, as indicated, vehicle (−) was set as 100% upon which the results induced by treatments was calculated. Each column represents the mean ± SD of two independent experiments, each performed in triplicate. (■) indicates *P* < 0.05 for cells receiving treatments versus vehicle (−)

In line with our previous studies showing that estrogenic GPER signaling induces proliferative effects in cancer cells and CAFs through growth regulatory genes like cyclins [47, 77, 80], we determined that 3MC stimulates the expression of cyclin D1 at both mRNA and protein levels in SkBr3 cells (Fig. 5a-c) and CAFs (Fig. 5a, d-e) through AHR and GPER as assessed using the inhibitors of these receptors, CH223191 and G15, respectively. On the basis of previous data indicating that CYP1B1 is involved in growth responses [19, 47], we then determined that the CYP1B1 inhibitor 2,4,3′,5′-tetramethoxystilbene (TMS) as well as the silencing of CYP1B1 expression prevent the up-regulation of cyclin D1 by 3MC in SkBr3 cells (Fig. 5f-h) and CAFs (Fig. 5j-l). The Specificity Protein 1 (SP1) transcription factor has been shown to contribute to the CYP1B1 mediated increase of growth regulatory genes like cyclin D1 as well as the proliferation, migration and invasion of cancer cells [19, 81–85]. On the basis of these findings and considering the role elicited by SP1 toward the oncogenic transformation prompted by CYP1B1 [19], we next established that cyclin D1 protein induction by 3MC is abolished using the SP1 inhibitor MTM A in SkBr3 cells (Fig. 5i) and CAFs (Fig. 5m). Recapitulating the aforementioned results, the proliferation of two-dimensionally (2D)-cultured SkBr3 cells induced by 3MC was no longer evident either in the presence of CH223191, G15, TMS and MTM A, inhibitors respectively of AHR, GPER, CYP1B1 and SP1, or silencing CYP1B1 and GPER expression (Fig. 6a-h). Similar results were also obtained in a three-dimensional (3D)-culture system (Fig. 6i-j). Collectively, these findings suggest that AHR and GPER are involved in the CYP1B1 and cyclin D1 induction upon exposure to 3MC toward the growth responses observed in breast cancer cells and CAFs.

## Discussion

The environmental pollutant 3MC, which is present in cigarette smoke and generated by incomplete combustion processes, exerts carcinogenic effects mainly through the AHR [30, 33, 34, 78]. Likewise, several contaminants like dioxin, BaP and 7,12-dimethylbenz [*a*] anthracene (DMBA) trigger the transcription of pro-carcinogenic genes binding to and activating AHR toward cancer cell proliferation, invasion and drug resistance [22, 27, 68, 78]. In addition, the ligands of AHR may induce their own metabolism regulating the expression and the activity of drug-metabolizing cytochrome P450 enzymes as CYP1B1 [23, 78, 83, 86], leading to the formation of nucleophilic derivatives and epoxides that increase the risk of carcinogenesis [13, 14]. It is worth mentioning that high levels of CYP1B1 may increase the expression of AHR, therefore triggering a feed-forward loop that facilitates tumor progression [87].

**Fig. 7:**
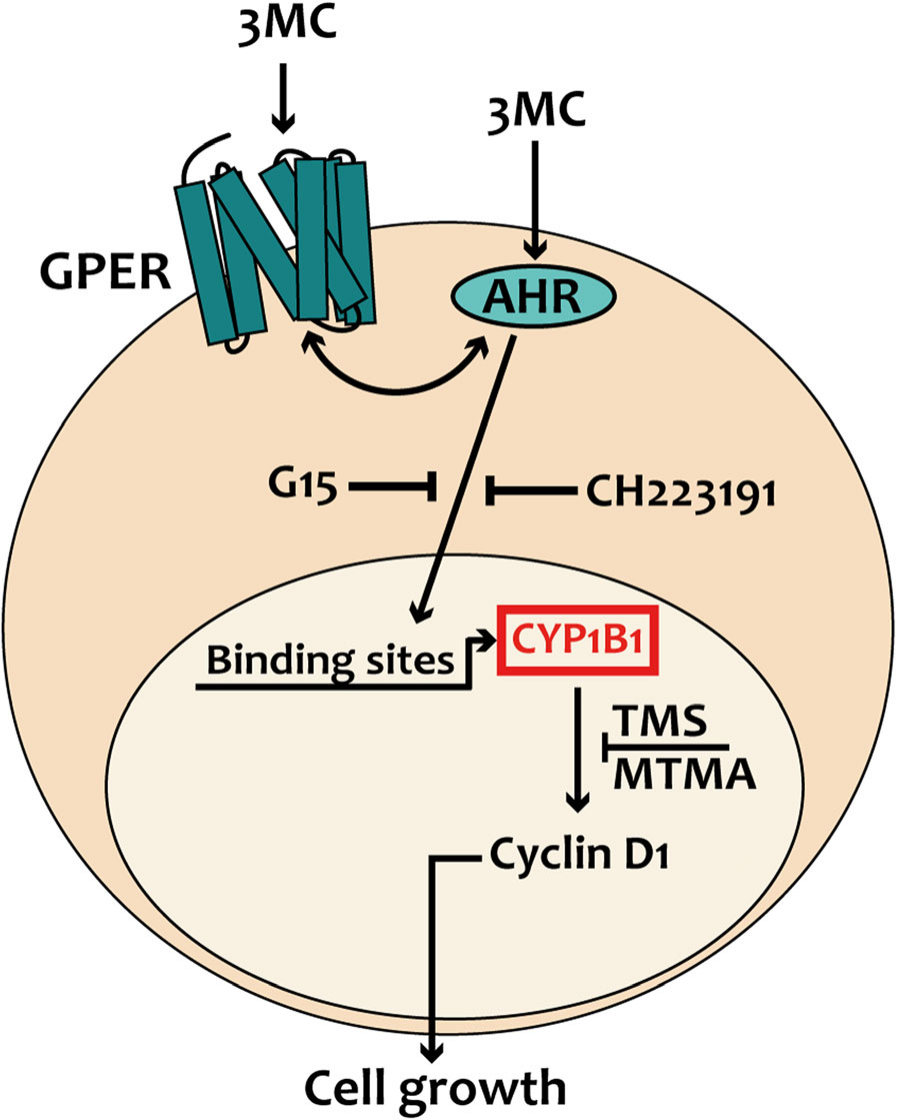
Schematic representation of CYP1B1 regulation by both AHR and GPER signaling

Diverse studies have demonstrated that 3MC and other PAHs could act as ERα ligands and activators, thus exhibiting the ability to regulate certain estrogen target genes in hormone-dependent tumors [31, 32, 78]. Although the effects elicited by both natural and synthetic estrogens in cancer cells are typically mediated by ERα, previous investigations have also ascertained a role for GPER [35, 39, 40]. In this regard, we have recently demonstrated the involvement of GPER in the estrogen regulation of CYP1B1 that, in turn, prompted growth effects in diverse breast tumor models [47]. Further extending these data, the current results demonstrate that 3MC engages GPER toward the up-regulation of both CYP1B1 and the cell-cycle regulator cyclin D1 in breast tumor cells and major components of the cancer stroma as CAFs **(**Fig. 7**)**.

In the absence of ligands, AHR forms a cytosolic protein complex together with the molecular chaperone heat shock protein 90 (HSP90), the co-chaperone protein p23, the hepatitis B virus X-associated protein 2 (XAP2) and the Src tyrosine kinase [88]. The agonist binding to AHR promotes the translocation into the nucleus of a heterodimer formed by the receptor along with the aryl hydrocarbon receptor nuclear translocator (ARNT), which belongs to the same AHR family named bHLH-PAS (basic Helix-Loop-Helix – Period/ARNT/Single minded) [88]. The AHR/ARNT complex regulates gene transcription interacting with specific response elements located in the promoter sequences of target genes as CYP1B1 [15, 22, 70, 83, 89]. In this vein, we have ascertained that the nuclear translocation of AHR induced by 3MC is abrogated in the presence of the AHR inhibitor CH223191 and the GPER antagonist G15 in breast cancer cells. Nevertheless, a better assessment on the role of GPER in the nuclear shuttle of AHR remains to be elucidated in next studies.

The stimulatory responses elicited in cancer cells by ligands of AHR like 3MC may involve a crosstalk of AHR with diverse signaling molecules and transduction pathways as EGFR [70, 71], the transforming growth factor-β (TGF-β) and tumor necrosis factor-α (TNF-α) [90]. This cooperative action, which promotes the dissociation of AHR from the cytosolic chaperone complex and its subsequent nuclear translocation, may have important implications toward the development of various malignancies including breast cancer [27, 70, 88, 91, 92]. In particular, a Src-mediated crosstalk between AHR and EGFR has been shown to trigger the activation of ERK1/2 and the stimulation of proliferative effects in cancer cells [70, 71]. In accordance with these findings and previous studies showing that the association of GPER with growth factor receptors as EGFR and IGF-IR may activate transduction signals in cancer cells [75, 77], we have determined that 3MC triggers a physical association of GPER with AHR and EGFR leading to their functional interaction in breast cancer cells. Altogether, our results indicate that GPER is involved in a transduction network that includes AHR and EGFR toward 3MC-induced stimulatory effects in breast cancer cells.

Clinical observations have indicated that GPER expression may be associated with negative clinical features and poor survival rates in diverse types of malignancies [93, 94], although other studies have reported opposite results [95–98]. Numerous investigations have also shown that GPER mediates estrogenic signaling in several tumors including breast cancer [39, 46, 94, 99]. For instance, it has been demonstrated that ligand-activated GPER triggers EGFR transactivation and subsequent transduction events such as the activation of MAPK and PI3K, gene transcription and biological responses like proliferation, migration and angiogenesis in breast cancer cells and CAFs [45]. On the basis of the potential role elicited by GPER in tumor progression, several studies have been performed in order to identify GPER ligands that may promote relevant activities in tumor cells [69, 80, 100–105]. In this regard, the environmental contaminant bisphenol A [40] and the pesticide atrazine [39] were shown to trigger stimulatory effects through GPER in breast cancer cells and CAFs. Further searching for the ligand activation of GPER signaling, we have here ascertained that 3MC may also engage GPER leading to the activation of the EGFR/ERK/c-Fos transduction pathway and the increase of both CYP1B1 and cyclin D1 expression. Noteworthy, the proliferative effects triggered by 3MC were prevented through the inhibition of AHR and GPER in our model system, thus suggesting that both receptors may be involved in the intricate actions of this important environmental pollutant.

## Conclusions

The ligand-activated transcription factor AHR, which is an important intracellular chemosensor responsive to environmental chemicals, plays a relevant role in the xenobiotic-induced carcinogenesis. Several compounds like aromatic hydrocarbons as well as estrogens can stimulate the expression of the AHR target gene CYP1B1, which is overexpressed in a variety of tumors including breast cancer. Here, we have provided novel insights on the crosstalk that may occur between AHR and GPER upon exposure to 3MC, toward the up-regulation of CYP1B1 and cyclin D1 expression as well as the proliferative effects observed in breast cancer cells and main components of the tumor microenvironment as CAFs.

## Additional file

Additional file 1: CAFs characterization*.* CAFs were immunostained by anti-FAPα, anti-Vimentin and anti-Cytokeratin14 antibodies. Green signal: FAPα; Red signal: Vimentin; Blue signal: Nuclei. Scale bar: 200 μm. (DOC 1149 kb)

## Data Availability

Data sharing not applicable to this article as no datasets were generated or analysed during the current study.

## Abbreviations

3MC: 3-methylcholanthrene
AHR: Aryl Hydrocarbon Receptor
ARNT: Aryl hydrocarbon receptor nuclear translocator
CAFs: Cancer-associated fibroblasts
cAMP: cyclic AMP
CYP1B1: Cytochrome P450 1B1
E2: 17β-Estradiol
EGFR: Epidermal Growth Factor Receptor
ER: Estrogen Receptor
G-1: [1,3] diodo-5-yl)-3a,4,5,9b-tetrahidro-3H-5-cyclopenta [c]quinolin-8yl]-ethanone)
G15: (3aS,4R,9bR)-4-(6-bromo-1,3-benzodioxol-5-yl)-3a,4,5,9b-3H-cyclopenta [c]quinolone
GPER: G protein-coupled estrogen receptor
HSP90: Heat shock protein 90
MAPK: Mitogen-activated protein kinase
MD: Molecular dynamics
MTM A: Mithramycin A
PAHs: Policyclic aromatic hydrocarbons
SP1: Specificity Protein 1
TIS: Transcription initiation site
TMS: 1-[2,(3,5-dimethoxyphenyl) ethenyl]-2,4-dimethoxybenzene (2,4,3′,5′-tetramethoxystilbene)
XAP2: Hepatitis B virus X-associated protein 2
