# Supplementary material for: AHR and GPER mediate the stimulatory effects induced by 3-methylcholanthrene in breast cancer cells and cancer-associated fibroblasts (CAFs)
Source: J Exp Clin Cancer Res. 2019 Aug 1;38:335. doi: 10.1186/s13046-019-1337-2 (PMC6676524; doi:10.1186/s13046-019-1337-2)
Supplement: Supplementary file 1 — CAFs characterization. CAFs were immunostained by anti-FAPα, anti-Vimentin and anti-Cytokeratin14 antibodies. Green signal: FAPα; Red signal: Vimentin; Blue signal: Nuclei. Scale bar: 200 μm. (DOC 1149 kb) [file 13046_2019_1337_MOESM1_ESM.doc]

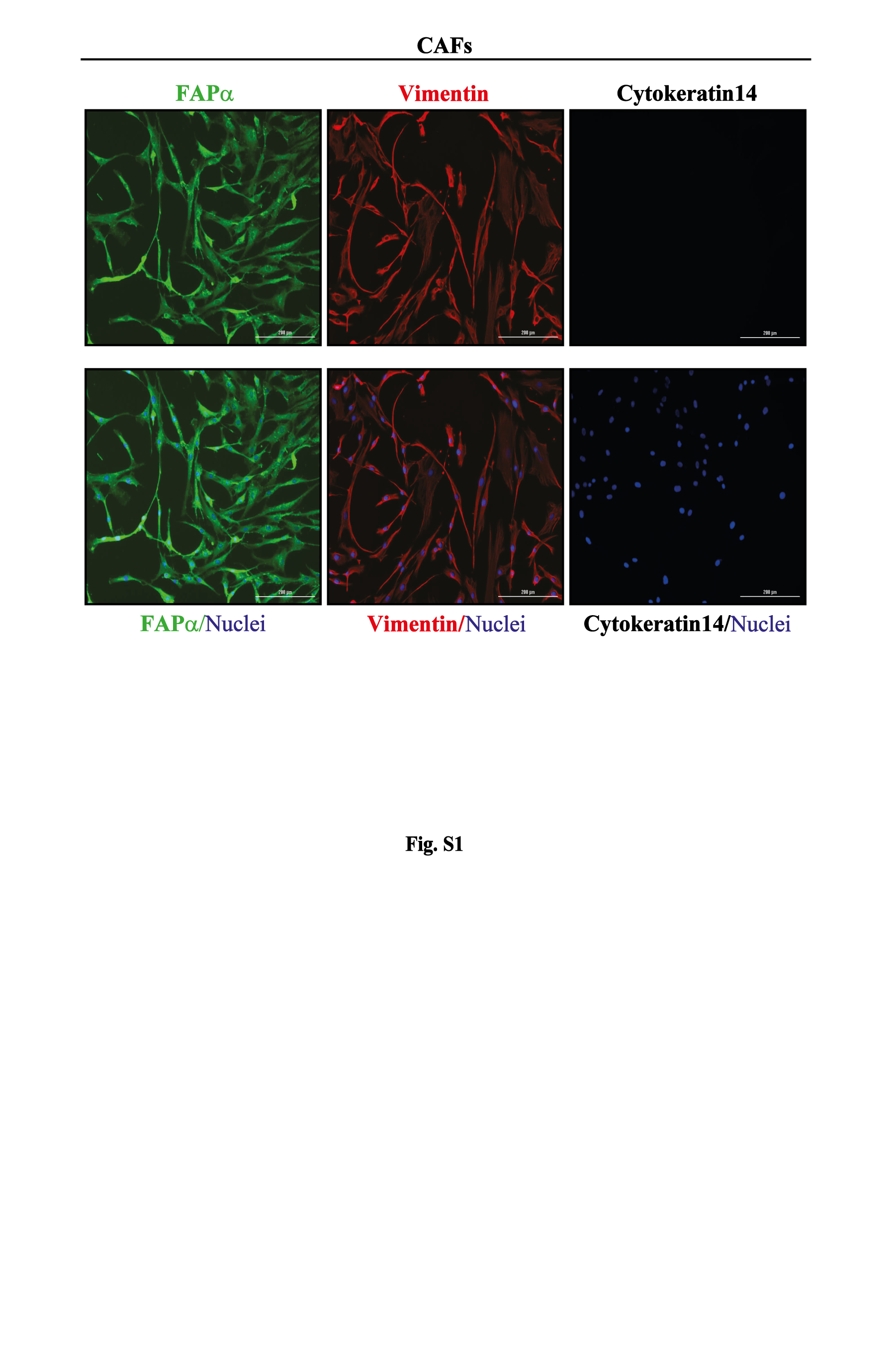


**Additional file 1. CAFs characterization*.*** CAFs were immunostained by anti-FAPα, anti-Vimentin and anti-Cytokeratin14 antibodies. Green signal: FAPα; Red signal: Vimentin; Blue signal: Nuclei. Scale bar: 200 μm.
